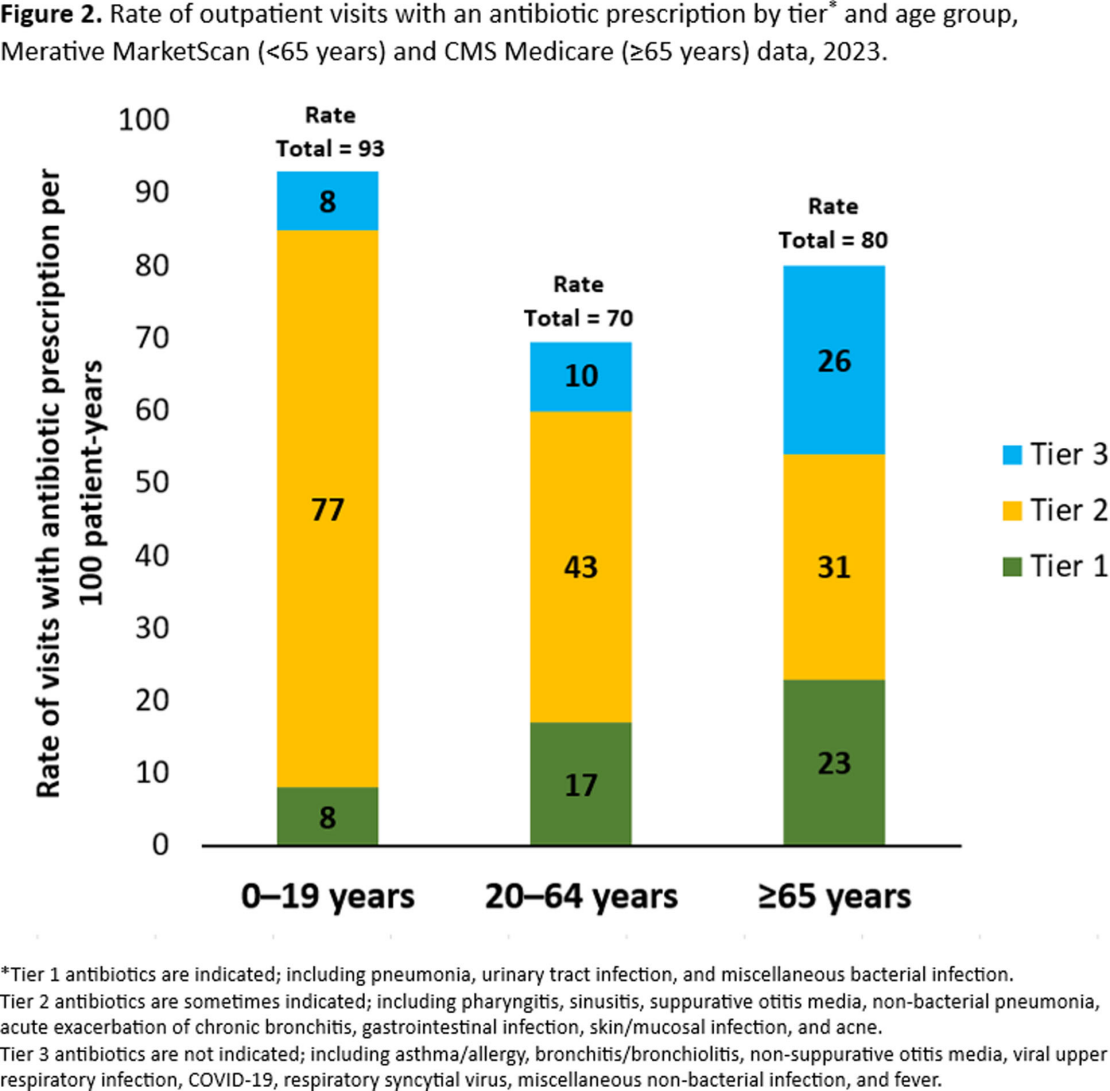# 79 Urinary Tract Infection Treatment Guidelines vs. resistance in Poland: Epidemiologic Insights from Southern Poland

**DOI:** 10.1017/ash.2026.10507

**Published:** 2026-06-23

**Authors:** Madeline Powers, Emily McDonald, Christine Kim, Tessa Schwarze, Lauri Hicks, Adam Hersh, Sarah Kabbani

**Affiliations:** 1 CDC Foundation; 2 CDC/DHQP; 3 CDC; 4 Chenega Corporation; 5 Centers for Disease Control and Prevention; 6 University of Utah

## Abstract

**Background:** Previous studies have shown that unnecessary antibiotic use is common in outpatient settings, particularly for acute respiratory illnesses. We assessed changes in outpatient antibiotic prescribing and estimated the proportion of unnecessary prescribing to identify additional opportunities for improvement and evaluate progress toward national goals to improve antibiotic use. **Methods:** We used Merative MarketScan commercial claims (patients aged 0-19 and 20-64 years) and Centers for Medicare & Medicaid Services (CMS) Medicare carrier claims and Part D event files (patients aged ?65 years) to identify enrollees with medical and prescription drug coverage for 2019 and 2023. Enrollees were weighted by months of enrollment. Using ICD-10 codes, outpatient encounters were assigned to a single diagnosis using a tiered algorithm based on the most likely indication for antibiotics. Tier 1 included diagnoses for which antibiotics are indicated (e.g., pneumonia, urinary tract infection); tier 2 included diagnoses for which antibiotics are sometimes indicated (e.g., sinusitis, pharyngitis, suppurative otitis media); and tier 3 included diagnoses for which antibiotics are not indicated (e.g., bronchitis, viral upper respiratory infection). Oral antibiotics dispensed on the visit date or within 7 days afterward were linked to the encounters. We calculated the proportion and rate (per 100 patient-years) of antibiotic-associated encounters by tier and age group and reported percent change from 2019 compared to 2023. **Results:** The proportion of adult outpatient encounters resulting in an antibiotic prescription was lower in 2023 compared to 2019 across diagnostic tiers (Figure 1). The largest reduction occurred for tier 3 conditions, where antibiotic-associated encounters decreased by 32% among adults aged ?65 years and by 30% among adults aged 20-64 years. Tier 3 prescribing was highest for adults aged ?65 years. In 2023, the highest antibiotic prescribing rates were for tier 2 conditions, particularly among children aged 0-19 years (77 antibiotics/100 patient-years) and adults aged 20-64 years (43 antibiotics/100 patient-years) (Figure 2). **Conclusions:** Using nationally representative data, we found that adults had fewer healthcare visits associated with antibiotic prescriptions in 2023 compared with 2019, with the largest declines occurring for conditions for which antibiotics are unnecessary. However, there are still opportunities to reduce unnecessary antibiotic prescriptions, especially for older adults. Antibiotic stewardship efforts should also focus on conditions for which antibiotics are sometimes indicated (e.g., sinusitis). This includes assessing diagnosis-specific criteria for prescribing an antibiotic and evaluating antibiotic selection and duration to benchmark appropriateness and tailor improvement efforts.

**Figure:**
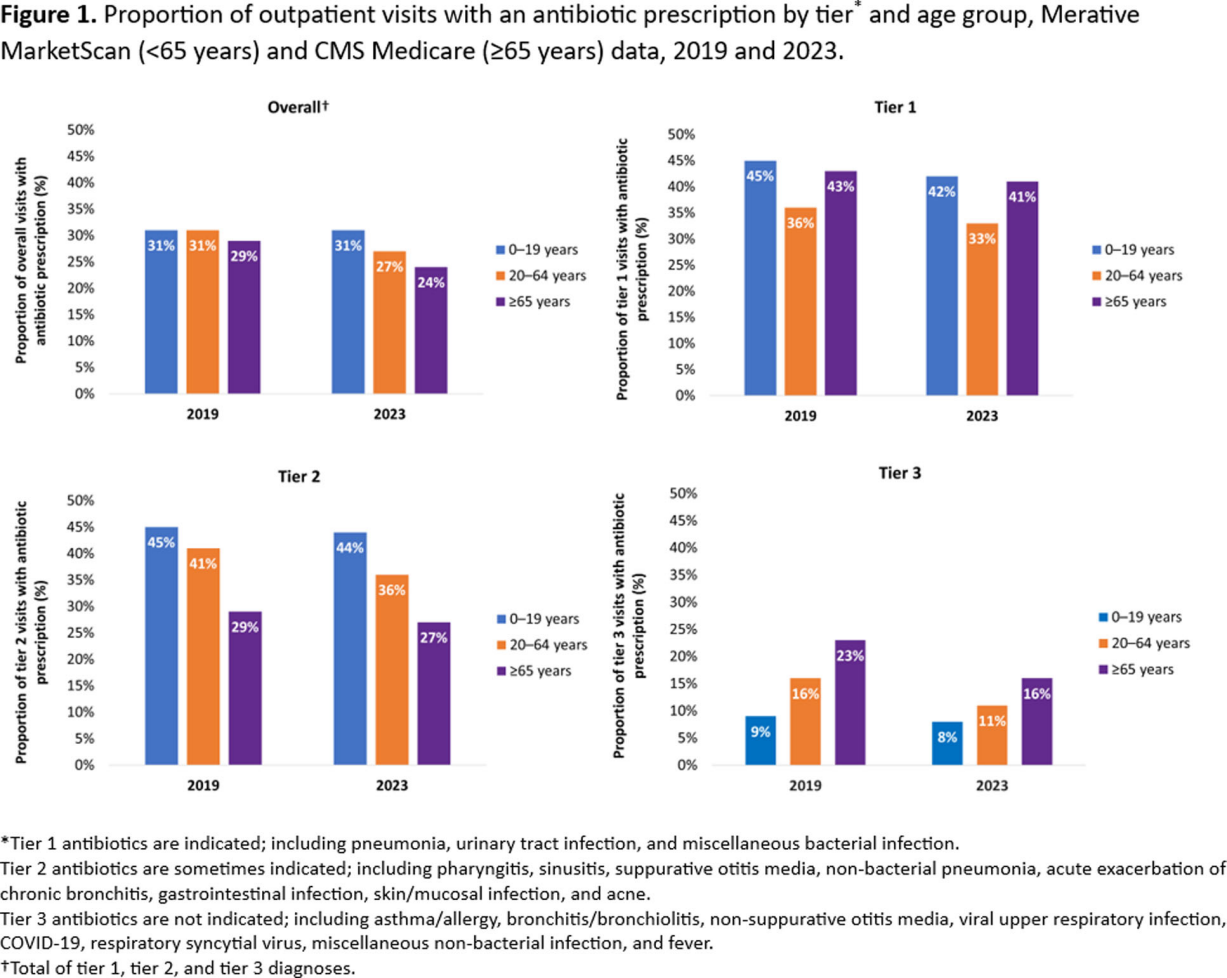

**Figure:**